# Spontaneous hemispheric ventricular collapse and subarachnoid haemorrhages in a dog with congenital hydrocephalus internus

**DOI:** 10.1186/s13620-020-00159-x

**Published:** 2020-03-25

**Authors:** Agnieszka Olszewska, Daniela Farke, Martin Jürgen Schmidt

**Affiliations:** Department of Veterinary Clinical Sciences, Small Animal Clinic, Neurosurgery, Neuroradiology and Clinical Neurology, Justus-Liebig-University, Frankfurter Strasse 108, 35392, Giessen, Germany

**Keywords:** Brain herniation, Canine, Internal hydrocephalus, Intracranial bleeding, Magnetic resonance imaging

## Abstract

**Background:**

Overdrainage and collapse of the hemispheres is a potential severe complication after surgical treatment of internal hydrocephalus using ventriculoperitoneal shunts. Here we describe a case of a spontaneous hemispheric ventricular collapse in an untreated dog with congenital hydrocephalus internus.

**Case presentation:**

A twelve-week-old, male, intact Golden Retriever was presented with a history of peracute obtundation, impaired vision, and progressive gait abnormalities of all limbs for three days. Neurological examination revealed a dome shaped skull, a broad-based stance and a moderate cerebellar ataxia. The postural responses were markedly delayed in all limbs. Moderate ventro-lateral strabismus, vertical nystagmus and absent menace response were observed bilaterally. Clinical signs indicated multifocal localisation (forebrain, cerebellum). Magnetic resonance imaging (MRI) showed dilation of all cerebral ventricles, irregular thinning of the periventricular white and grey matter, consistent with internal hydrocephalus. In addition, the hemispheres were collapsed at the right temporal and left frontal lobe with haemorrhage filling the adjacent subarachnoid space. The dog underwent left frontal and right temporal craniotomy for removal of the haemorrhage. The dog improved on all neurological signs and was discharged after seven days. A repeat MRI three months postsurgical intervention showed reexpansion of the cerebral hemispheres. Subarachnoid haemorrhages were markedly reduced.

**Conclusions:**

Collapse of the hemispheres can occur spontaneously in dogs with hydrocephalus internus. Removal of the haemorrhage can improve clinical signs.

## Background

Internal hydrocephalus, defined as an accumulation of the cerebrospinal fluid (CSF) within the ventricular system of the brain, is the most common congenital anomaly of the nervous system in dogs [1–3]. Progressive dilation of the ventricles is caused by an imbalance between production and absorption of CSF, which causes subsequent increase in intraventricular and intracranial pressure and the development of the hypertensive hydrocephalus [4–6]. The increased intracranial pressure in dogs with hydrocephalus causes progressive compromise of cerebral vessels, lacerations of the periventricular white matter, focal destruction of the ependymal lining and can lead to permanent neuronal injury and severe white matter atrophy [2, 7, 8]. Overdrainage and collapse of the hemispheres is a potential severe complication after surgical treatment of internal hydrocephalus using ventriculoperitoneal shunts in humans [9, 10] and animals [3, 11]. The development of subarachnoid haemorrhages and hematomas secondary to hemispheric ventricular collapse leads to acute progression of neurological signs and decompressive surgery is the first choice of treatment in human patients [12, 13] and animals [14].

To the author’s knowledge, this is the first case of a spontaneous hemispheric collapse in an untreated dog with congenital internal hydrocephalus. Here, we report successful surgical treatment of subarachnoid haemorrhages secondary to spontaneous hemispheric ventricular collapse in a dog with congenital hydrocephalus internus.

## Case presentation

A twelve-week-old, male, intact Golden retriever was presented for investigation of peracute obtundation, impaired vision, and gait abnormalities of all limbs. According to the owner, no traumatic insult was observed. General physical examination was unremarkable. Neurological examination revealed moderate obtundation, a dome shaped skull, a broad-based stance and a moderate cerebellar ataxia with hypermetria on the forelimbs. The postural responses were markedly delayed in all limbs. Moderate ventro-lateral strabismus, positional vertical nystagmus and absent menace response were observed bilaterally. The visual tests including cotton ball test and visual positioning were delayed to absent. There was marked pain reaction by palpation of the cervical spine region. The findings were consistent with an intracranial multifocal localisation with suspected involvement of forebrain and cerebellum. The main differential diagnoses were congenital anomalies, inflammatory, toxic and metabolic diseases. Preanesthetic laboratory investigations comprising complete blood cell count (CBC), serum biochemistry panel and electrolytes were unremarkable with the exception of a mildly elevated creatine kinase (392 U/L; reference range, 10–143 U/L), alkaline phosphatase (371 U/L; reference range 0–130 U/L), calcium (1.59 mmol/L; reference range 1.23–1.43 mmol/L), and phosphate (2.85 mmol/L; reference range 0.79–2.1 mmol/L), attributed to the young age of the dog.

The dog was initially stabilised with fluid therapy (crystalloid solution, 2 mL/kg, Sterofundin®, Braun) and underwent premedication using diazepam (0.5 mg/kg i.m, Ziapam, 5 mg/mL, solution for injection, TVM, UK). General anesthesia was induced with propofol (4–8 mg/kg i.v, Vetofol, emulsion for injection 1.0%, Norbrook) and maintained by inhaled anesthetics (isoflurane and oxygen mixture; 1.5% volume/volume; oxygen flow, 2 L/min).

MRI of the brain was acquired in ventral recumbency with 3 Tesla scanner (Siemens, Magnetom Verio). The following MRI sequences were applied: Fast spin echo (FSE) T2-weighted (T2W) sagittal, dorsal and transverse images, T2W fluid attenuated inversion recovery (FLAIR) transverse images, transverse gradient echo images (susceptibility weighted imaging-SWI), and FSE T1-weighted (T1W) 3D images following intravenous administration.

**Fig. 1:**
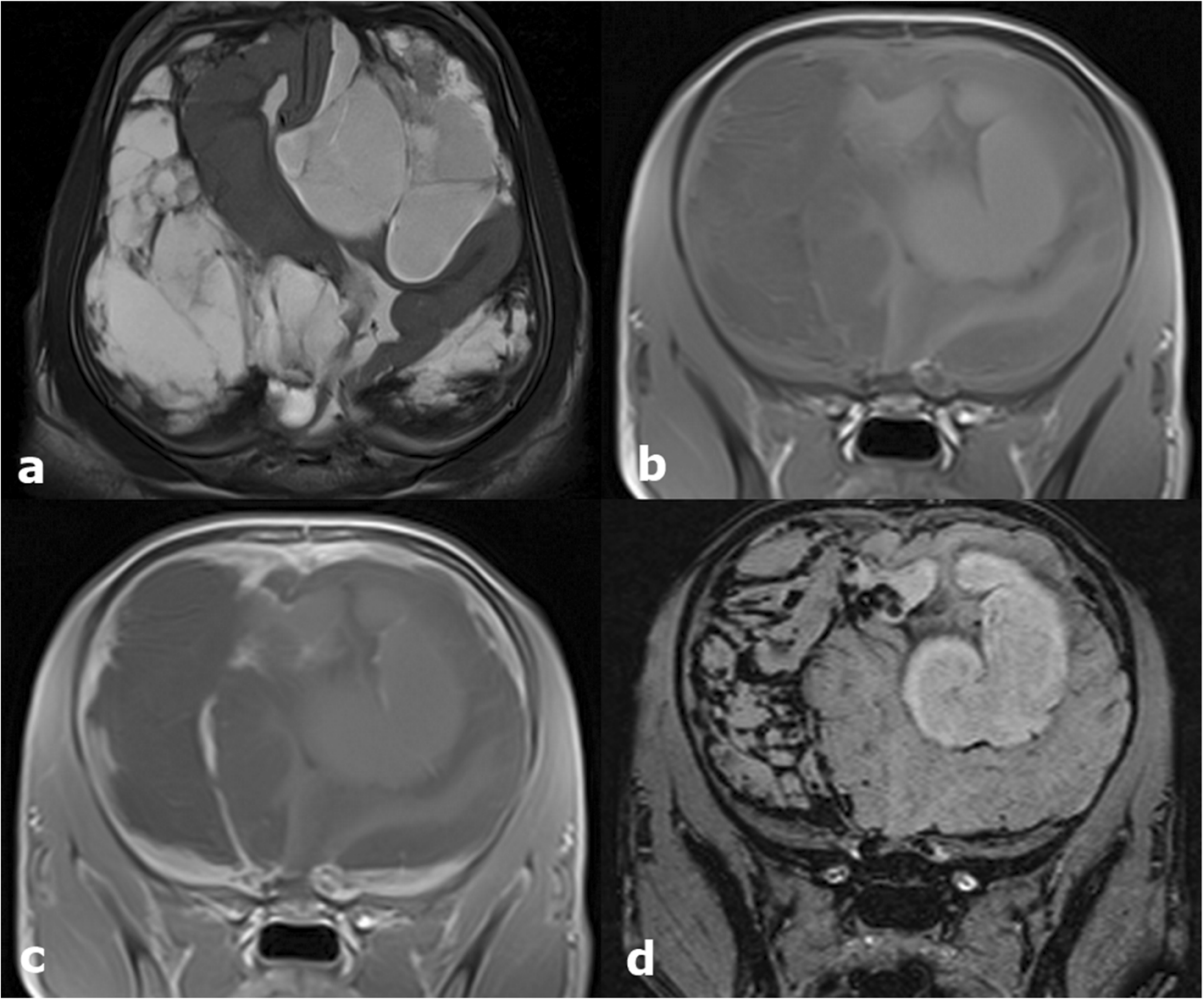
MRI images of the twelve-week-old, male Golden Retriever with internal hydrocephalus and acute ventricular collapse. Dorsal T2W (**a**), transverse T1W (**b**), transverse T1W after contrast administration (**c**) and transverse SWI (**d**) images of the brain. MRI showed marked thinning of the cerebral parenchyma and collapse of the hemispheres with marked subacute and chronic haemorrhage filling the adjacent subarachnoid space extending from the frontal lobe to the occipital lobe bilaterally. In the SWI sequence (**d**), the material presented multifocal susceptibility artifacts confirming the presence of the haemorrhages

MRI showed domed shaped calvaria with evidence of an asymmetric conformation of the frontal bones with focal thinning of the right frontal bone. The parenchyma of the cerebral hemispheres was markedly thinned. At the level of the left frontal lobe there was a marked, heterogenous, lobulated lesion in the subarachnoid space with a severe mass effect on the cortical surface causing medial displacement of the brain parenchyma. From the frontal lobe caudally towards the occipital bone, there was an comparable broad-based lesion with severe mass effect on the hemisphere that was deformed and displaced towards the right side. The ventricular system was entirely collapsed at the right temporal and left frontal lobe. A third lesion was seen between the occipital lobes and the cerebellum which created caudal displacement and herniation of the caudal aspect of the cerebellum through the foramen magnum. The material between brain surface and meninges was characterized by severe heterogeneous hyperintense signal in T2W/FLAIR and heterogenous isointense T1W with moderate peripheral and internal contrast uptake (Fig. 1a-d). In the gradient echo sequence, the material presented multifocal susceptibility artifacts. MRI was consistent with internal hydrocephalus. In addition, the hemispheres were collapsed at the right temporal and left frontal lobe with subacute and chronic haemorrhage filling the adjacent subarachnoid space as well as subtentorial and cerebellar herniation.

Based on the MRI findings, blood sample for the coagulation profile including prothrombin time (PT), partial thromboplastin time (PTT), antithrombin III, fibrinogen and D-dimers was evaluated. The results were unremarkable. Because of marked signs of increased intracranial pressure on the MRI, cerebrospinal fluid puncture was not performed at that point.

Due to the severity of neurological signs including obtundation and the presence of marked signs of increased intracranial pressure, the dog was taken to emergency surgery for decompression. Left frontal and right temporal craniotomy for removal of the haemorrhage was performed. The dural surface was bulging over the craniotomy, indicating high intracranial pressure The subarachnoid haemorrhages were evacuated gradually. Twenty milliliters of brownish, serous fluid were punctured and submitted for cytology and bacterial culture. Normal CSF pulsation was observed after removing the fluid. Following craniotomy, suboccipital decompressive craniectomy was obtained to minimize cerebellar herniation.

While the results of cytology and bacterial culture were pending, intravenous antibiotic treatment with cefotaxime (20 mg/kg i.v; Hexal, 1 g, powder for solution for injection, Fresenius Medical Care, Germany) was initiated to minimize the risk of postoperative sepsis and was continued over seven days. Postoperative care included electrocardiography (ECG), supplementation of oxygen, frequent blood pressure control and blood gas analysis with electrolyte status, fluid therapy (isotonic saline solution, 0.9%, 4 mL/kg, B. Braun, Melsungen AG) and pain management with metamizole (50 mg/kg p.o., Hexal®, Fresenius Medical Care, Germany), gabapentin (10 mg/kg p.o., Sandoz® Pharmaceuticals GmbH) and methadone (1 mg/kg i.v, Comfortan® 10 mg/mL solution for injection for dogs and cats, Dechra Veterinary Products, UK) and strict cage rest. In the meantime, results of the bacterial culture were available and were unremarkable. Venous blood gas analysis with electrolytes and complete blood work were repeated, with no abnormal results. The dog’s neurological status improved over the following days showing bright mentation and ambulatory gait with no proprioceptive deficits. Moderate cerebellar ataxia with moderate visual impairment was further observed. The dog was discharged from the hospital after seven days.

In the follow-up neurological examination three months after the initial consult, the dog showed complete resolution of mentation deficits and gait abnormalities. Moderate ventro-lateral strabismus was still present. The menace response was moderately reduced bilaterally, however previously documented visual deficits were not noticed any longer.

**Fig. 2:**
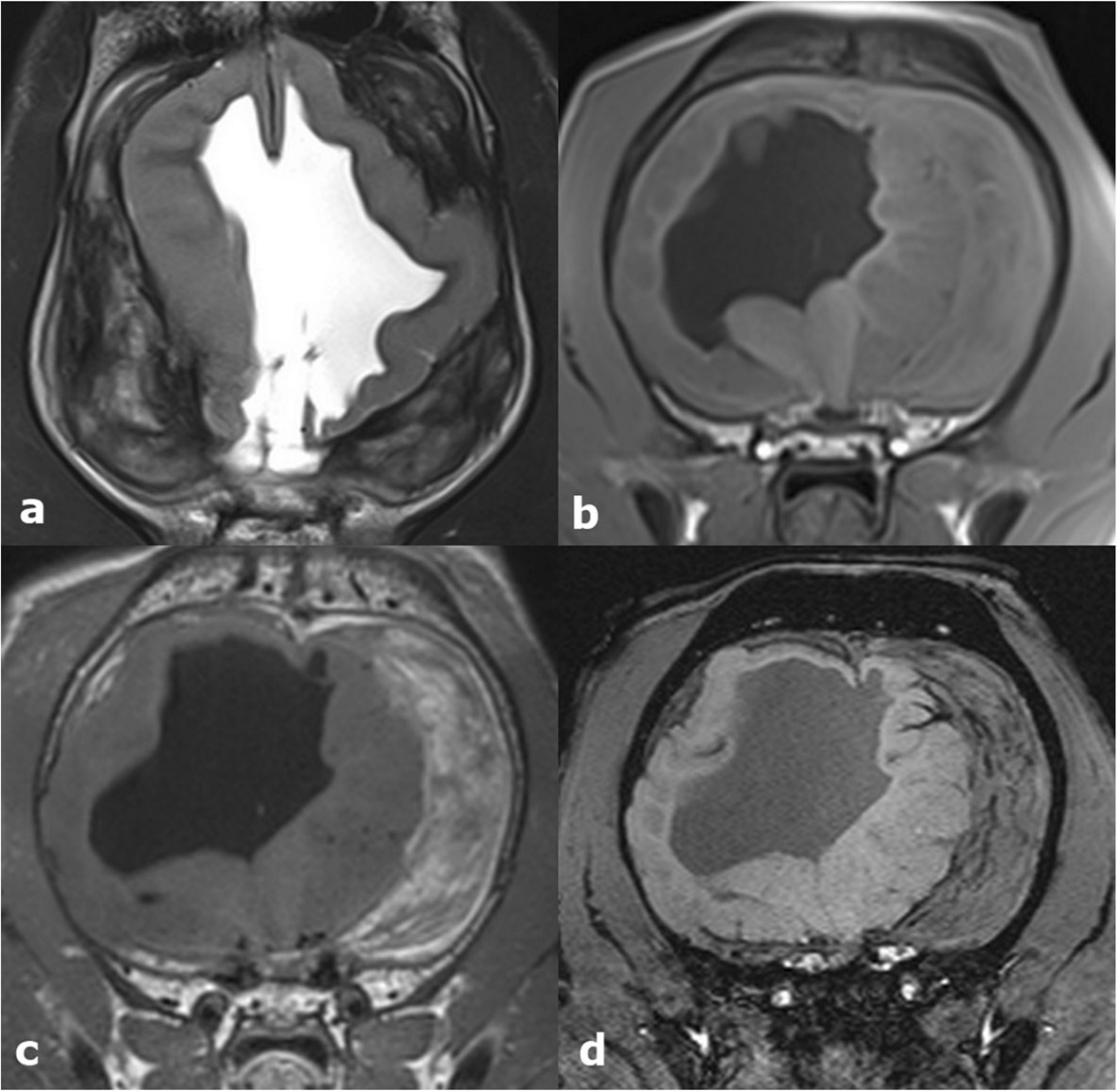
Follow-up MRI three months postsurgical intervention of the Golden Retriever with ventricular collapse. Dorsal T2W (**a**), transverse T1W (**b**), transverse T1W after contrast administration (**c**) and transverse SWI (**d**) images of the brain. Repeated MRI showed an increase in volume of the cerebral parenchyma and a moderate reduction in volume of the haemorrhages in comparison to previous examination

The repeat MRI showed an increase in volume of the cerebral parenchyma in comparison to previous examination. The subarachnoid haemorrhages were significantly decreased in size, but still present. Their signal intensity changed compared to previous exam, being characterized by severe heterogeneous mixed hypointense and hyperintense signal in T2W/FLAIR, isointense in T1W with moderate peripheral and internal contrast uptake (Fig. 2 a-d). There was moderate mass effect on the cerebellum which showed reduction in volume, irregular shape with cranial flattening. The cerebellar parenchyma showed mild increase in volume. Minimal herniation of the caudal aspect of the cerebellum through the craniectomy site was present. The ventricular system was asymmetric and moderately distended, the mesencephalic aqueduct could not be delineated, which suggests aquaeductal stenosis as the underlying cause for the internal hydrocephalus. In follow up clinical assessments after six and twelve months postsurgical examination, the neurological status of the dog was maintained, therefore no further imaging was obtained.

## Discussion

Neurological signs in dogs and cats with congenital internal hydrocephalus usually occur within the first 6 months of life [5]. Commonly observed clinical sings indicate mostly forebrain disorder- visual impairment, obtundation, circling and behavioral abnormalities [5, 15, 16]. The majority of dogs show a slow progression of neurological deficits. Occasionally, acute worsening of the clinical status is observed, usually following a minor traumatic event [5]. To our knowledge, this is the first case report of spontaneous hemispheric ventricular collapse and secondary subarachnoid haemorrhage in an untreated dog with internal hydrocephalus. Ventricular collapse has been described as a consequence of an excessive shunting in patients with hydrocephalus internus after the surgical placement of the ventriculoperitoneal shunt (VPS) [3, 11]. Excessive shunting and associated ventricular collapse with occurrence of subdural hematomas or subarachnoid haemorrhages is a potential post-operative complication was found in 2.7–2.8% dogs [3, 11]. Current literature pertaining to veterinary medicine does not document any cases involving spontaneous ventricular collapse in dogs. Hydrocephalus rarely remains untreated in humans, therefore limited data is available on spontaneous ventricular pathology. One of the observed complications is the ventricular rupture secondary to intracranial hypertension in patients with untreated hydrocephalus [17, 18]. Other possible reason is the instability of intracranial pressure (ICP) within the ventricular system due to changes in pressure gradients. Gradient-related results in literature regarding studies on humans [19–21] and experimental studies on animals [22, 23] are contradictory. One of the hypothesis is that pressure gradients exist between the ventricles, brain tissue, and subarachnoid space when acute hydrocephalus develops [20, 21, 24, 25]. However, in an experimental study on six dogs using implant pressure sensors, no consistent pressure differences were found between the ventricle, brain, and subarachnoid space before kaolin administration or when hydrocephalus later developed [22]. It remains unclear, whether low/high pressure gradients, a sudden pressure decrease or reversed pressure gradients in the course of ventricular distension could cause sudden increase/decrease in ICP and spontaneous ventricular collapse in the present study. Studies on a larger group of dogs with internal hydrocephalus are necessary to demonstrate those mechanisms in animals.

Another theory regarding sudden change in the intracranial pressure is the head and body position. The effect of the head and body position and changes in the mean ICP have been recently examined using subdural and intraparenchymal miniature strain-gauge transducers in healthy dogs [26]. Head down positioning was correlated with an increase in ICP. Conversely, another report showed no correlation between the head position and ICP [27]. The actual cause of the ventricular collapse in the present case remains elusive.

The incidence of subdural hematomas and subarachnoid haemorrhages is a well described complication after shunt placement in human [12, 13, 28] and veterinary medicine [11, 29, 30] and are thought to be a result of stretching and eventual disruption of the dura-arachnoid desmosomal attachments and bridging veins while the intracranial pressure changes secondary to the placed shunt and excessing opening pressure in the valve [30, 31]. Sporadically, hematomas resolve spontaneously without surgical intervention. The dog showed clinical signs of sudden neurological deterioration, therefore the decision of the emergency evacuation of the subarachnoid haemorrhage and a decompressive craniectomy was made. The primary cause of the development of the haemorrhage in our case remains unclear. Although traumatic event has not been reported by the owner, it cannot be entirely excluded as a potential etiology. We speculate, that possibly changing intracranial pressure could have caused the rupture of the periventricular veins.

Neurological sings suggesting ventricular collapse in dogs with internal hydrocephalus can be non-specific and depend on the severity of the collapse. Acute onset neurological deterioration including change in mentation, sudden visual impairment with gait abnormalities including paresis and ataxia can be observed.

Emergency craniotomy for removal of subarachnoid haemorrhage and suboccipital craniectomy were performed to improve the neurological status of the dog and prevent further deterioration. The ventriculoperitoneal shunt was not placed in this case to avoid potential changes in intracranial pressure. In the follow-up examination, the neurological deficits almost completely resolved, improving dog’s quality of life so that no further surgical intervention was necessary.

## Conclusions

In conclusion, this report presents that ventricular collapse can occur spontaneously in dogs with severe congenital hydrocephalus internus. Removal of the subarachnoid haemorrhage as well as decompressive craniectomy is an effective treatment option in dogs suffering from secondary serious subarachnoid haemorrhage. Almost complete resolution of the acute neurological signs demonstrates an outstanding ability of the hydrocephalic cortex to function after extreme deformation.

## Data Availability

All data generated or analysed during this study are included in this published article.

## Abbreviations

CBC: Complete blood cell count
CSF: Cerebrospinal fluid
ECG: Electrocardiography
FLAIR: Fluid-attenuated inversion recovery
FSE: Fast spin echo
ICP: Intracranial pressure
MRI: Magnetic resonance imaging
PT: Prothrombin Time
PTT: Partial prothrombin time
SWI: Susceptibility weighted imaging
T1W: T1 weighted image
T2W: T2 weighted image
VPS: Ventriculoperitoneal shunt

## References

[1] Selby LA, Hayes HM, Becker SV (1979). Epizootiologic features of canine hydrocephalus. Am J Vet Res.

[2] Wünschmann A, Oglesbee M (2001). Periventricular changes associated with spontaneous canine hydrocephalus. Vet Pathol.

[3] Biel M, Kramer M, Forterre F, Jurina K, Lautersack O (2013). Outcome of ventriculoperitoneal shunt implantation for treatment of congenital internal hydrocephalus in dogs and cats: 36 cases (2001–2009). JAVMA..

[4] Coates JR, Axlund TW, Dewey CW, Smith J (2006). Hydrocephalus in dogs and cats. Compend Contin Educ Pract Vet.

[5] Dewey CW, Dewey CW (2003). Encephalopathies: disorders of the brain. A practical guide to canine and feline neurology.

[6] Rekate HL (2008). The definition and classification of hydrocephalus: a personal recommendation to stimulate debate. Cerebrospinal Fluid Res.

[7] James AE, Burns B, Flor WF (1975). Pathophysiology of chronic communicating hydrocephalus in dogs (Canis familiaris). Experimental studies. J Neurol Sci.

[8] Laubner S, Ondreka N, Failing K, Kramer M, Schmidt MJ (2015). Magnetic resonance imaging signs of high intraventricular pressure- comparison of findings in dogs with clinically relevant internal hydrocephalus and asymptomatic dogs with ventriculomegaly. BMC Vet Res.

[9] Hanak BW, Bonow RH, Harris CA, Browd SR (2017). Cerebrospinal fluid shunting complications in children. Pediatr Neurosurg.

[10] Niimura M, Takai K, Taniguchi M. Postoperative epidural haematomas associated with hydrocephalus caused by intraoperative overdrainage of cerebrospinal fluid: two case reports with a literature review of 19 cases. BMJ Case Rep. 2015. 10.1136/bcr-2014-206654. 10.1136/bcr-2014-206654 PMC4330440 25666241

[11] Gradner G, Kaefinger R, Dupré G (2019). Complications associated with ventriculoperitoneal shunts in dogs and cats with idiopathic hydrocephalus: a systematic review. J Vet Intern Med.

[12] Yamada SM, Tomia Y, Murakami H, Nakane M (2015). Management for traumatic chronic subdural hematoma patients with well-controlled shunt system for hydrocephalus. Clin Case Rep.

[13] Hoya K, Tanaka Y, Uchida T, Takano I, Nagaishi M (2011). Treatment of traumatic acute subdural hematoma in adult hydrocephalus patients with cerebrospinal fluid shunt. Clin Neurol Neurosurg.

[14] Shores A, Brisson B (2017). Current techniques in canine and feline neurosurgery.

[15] Vullo T, Manzo R, Gomez DG, Deck MDF, Cahill PT (1997). Diagnosis of cerebral ventriculomegaly in normal adult beagles using quantitative MRI. Vet Radiol Ultrasound.

[16] Thomas WB (2010). Hydrocephalus in dogs and cats. Vet Clin North Am Small Anim Pract.

[17] Pennybacker J, Russell DS (1943). Spontaneous ventricular rupture in hydrocephalus, with subtentorial cyst formation. J Neurol Neurosurg Psychiatry.

[18] Moghtaderi A, Rahimi-Movaghar V, Safdari M (2005). Spontaneous brain rupture: a complication of untreated hydrocephalus. Clin Neurol Neurosurg.

[19] Stephensen H, Tisell M, Wikkelsö C (2002). There is no pressure gradient in communicating or noncommunicating hydrocephalus. Neurosurgery..

[20] Conner ES, Foley L, Black PM (1984). Experimental normal-pressure hydrocephalus is accompanied by increased trans mantle pressure. J Neurosurg.

[21] Hakim S, Hakim C, Shapiro K, Marmarou A, Portnoy H (1984). A biomechanical model of hydrocephalus and its relationship to treatment. Hydrocephalus.

[22] Penn RD, Lee MC, Linninger AA, Miesel K, Ning Lu S (2005). Pressure gradient in the brain in an experimental model of hydrocephalus. J Neurosurg.

[23] Miše B, Klarica M, Seiwerth S, Bulat M (1996). Experimental hydrocephalus and hydromyelia: a new insight in mechanism of their development. Acta Neurochir.

[24] Levine DN (2008). Intracranial pressure and ventricular expansion in hydrocephalus: have we been asking the wrong question?. J Neurol Sci.

[25] Smillic A, Sobey I, Molnar Z (2005). A hydroelastic model of hydrocephalus. J Fluid Mech.

[26] Sturges BJ, Dickinson P, Tripp L, Udaltsova I, Lecouteur R (2018). Intracranial pressure monitoring in normal dogs using subdural and intraparenchymal miniature strain-gauge transducers. J Vet Intern Med.

[27] Bagley RS, Keegan RD, Greene SA, Harrington ML, Moore MP (1995). Pathologic effects in brain after intracranial pressure monitoring in clinically normal dogs, using a fiberoptic monitoring system. Am J Vet Res.

[28] Pudenz R, Foltz E (1991). Hydrocephalus: Overdrainage by ventricular shunts. A review and recommendations. Surg Neurol.

[29] Tani K, Tanga A, Itamoto K, Iwanaga T, Une S (2001). Hydrocephalus and syringomyelia in a cat. J Vet Med Sci.

[30] DeLahunta A, Glass E (2015). Meninges: subarachnoid space in: veterinary Neuroanatomy and clinical neurology.

[31] Samuelson S, Long DM, Chou SN (1972). Subdural hematoma as a complication of shunting procedures for normal pressure hydrocephalus. J Neurosurg.

